# Key Roles of Glutamine Pathways in Reprogramming the Cancer Metabolism

**DOI:** 10.1155/2015/964321

**Published:** 2015-10-25

**Authors:** Krzysztof Piotr Michalak, Agnieszka Maćkowska-Kędziora, Bogusław Sobolewski, Piotr Woźniak

**Affiliations:** ^1^Laboratory of Vision Science and Optometry, Faculty of Physics, Adam Mickiewicz University of Poznań, Umultowska Street 85, 61-614 Poznań, Poland; ^2^Nanobiomedical Center of Poznań, Umultowska Street 85, 61-614 Poznań, Poland; ^3^Department of Clinical Pharmacology, Chair of Cardiology, Poznań University of Medical Sciences, Długa Street 1/2, 61-848 Poznań, Poland; ^4^Polish Mother's Memorial Hospital-Research Institute, Outpatient Clinic, Rzgowska Street 281/289, Łódź, Poland

## Abstract

Glutamine (GLN) is commonly known as an important metabolite used for the growth of cancer cells but the effects of its intake in cancer patients are still not clear. However, GLN is the main substrate for DNA and fatty acid synthesis. On the other hand, it reduces the oxidative stress by glutathione synthesis stimulation, stops the process of cancer cachexia, and nourishes the immunological system and the intestine epithelium, as well. The current paper deals with possible positive effects of GLN supplementation and conditions that should be fulfilled to obtain these effects. The analysis of GLN metabolism suggests that the separation of GLN and carbohydrates in the diet can minimize simultaneous supply of ATP (from glucose) and NADPH_2_ (from glutamine) to cancer cells. It should support to a larger extent the organism to fight against the cancer rather than the cancer cells. GLN cannot be considered the effective source of ATP for cancers with the impaired oxidative phosphorylation and pyruvate dehydrogenase inhibition. GLN intake restores decreased levels of glutathione in the case of chemotherapy and radiotherapy; thus, it facilitates regeneration processes of the intestine epithelium and immunological system.

## 1. Introduction

The development of cancer therapy is the urgent aim for science today. Growing knowledge about the metabolism of cancer cells provides new interesting hints concerning the metabolic targeting of the treatment and searching for new drugs inhibiting the growth of cancer [1]. The current paper presents the review of recently developed biochemical aspects of cancer metabolism and possible use of this knowledge for targeting the therapy into mainstream metabolic enzymes. Main emphasis is placed on the possible effect of glutamine (GLN) supplementation as a nutrient supporting both the cancer growth and organism to fight against the cancer. The individual kinds of tumors are characterized by different metabolic alterations which determine possible positive or negative effect of GLN supplementation. In the current paper, different aspects of cancer metabolism are discussed and analyzed in this context.

The aim of this paper is to point especially to these aspects of GLN metabolism that could be positively used while planning the treatment of the cancer patient and to point to conditions that should be fulfilled in order to make the positive effects of GLN supplementation surpass the negative ones.

## 2. Metabolism of Glutamine

### 2.1. Metabolism of Glutamine in Healthy Cells

GLN is one of 20 amino acids, commonly existing in every protein. The mammal organism is able to synthesize it. GLN is a central point in the metabolism of majority of amino acids [2].

**Figure 1:**
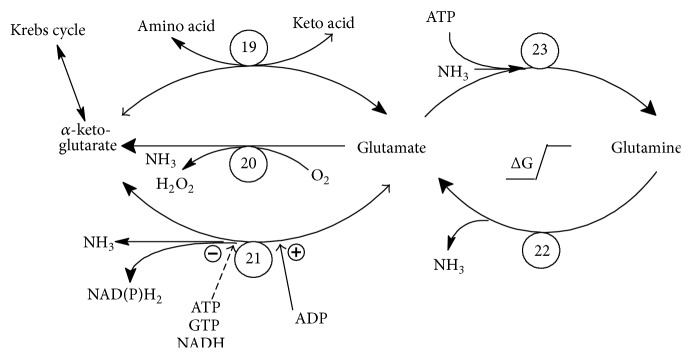
The connection between glutamine and tricarboxylic acid cycle: glutamine is metabolized to glutamate and next to *α*-ketoglutarate. Quick energy pathway from glutamine is open if glutamate dehydrogenase is activated by the low ATP and high ADP levels (as in majority of cancers). In the opposite, ATP and NAD(P)H_2_ are necessary for the synthesis of glutamine which takes place especially in muscles. Reactions 20, 21, and 22 are the source of ammonia and ammonia induced autophagy.* (Indices at enzyme abbreviations point to reaction numbers in Figures* 1 *and* 2.)

The first stage of amino acids metabolism consists in its change inside muscles tissue into the GLN and to lower degree into the alanine. The amino acids are metabolized to different tricarboxylic acid (TCA) cycle metabolites, enter TCA cycle, and go away as *α*-ketoglutarate (*α*KT) or alanine. *α*KT is next metabolized to GLN (Figure 1). Then, GLN is carried to other tissues like hepar, immunological system, intestinal tract, and fibroblasts where it is used up as the priority fuel [3, 4]. For this reason GLN comprises 60% of the total free amino acid pool in the blood plasma [5]. GLN synthesis rapidly decreases in the deficiency of energy in the cell. It occurs due to the fact that the energy from ATP and NADH_2_ has to be supplied to the synthesis of glutamine (Figure 1).

First, in Figure 1 let us analyze two reactions that metabolize reciprocal reactions between GLN and glutamate (GLU). These reactions are catalyzed by two different enzymes. It results from the relatively high free Gibbs energy between both substances. The decay of GLN occurs easily and with relatively high loss of free energy. In the opposite, the ATP is necessary to synthesize GLN. Thus, in the case of low ATP level in the cell, the equilibrium of this reaction is strongly shifted to the left (GLU creation).

The relation between *α*KT and GLU is more complicated. Three enzymes are capable of catalyzing this reaction [4]. Transaminase (TA^19^) carries the amine group from other amino acids into the *α*KT and it makes it possible to transfer the nitrogen into the urine cycle. The l-amino acid oxidase (AAO^20^) is the enzyme that acts only in one direction. It catalyses the deamination of GLU and produces the ammonia for urine cycle. The hydrogen peroxide that is generated in this reaction is quickly decayed by catalase and it makes this reaction irreversible. This reaction is characterized by the significant loss of free energy, as well. The third, crucial enzyme is the glutamate dehydrogenase (GDH^21^). This reaction is characterized with *K*_eq_ ≈ 1. It means that the direction of the reaction depends on the metabolic state of the organism. It should be noted that the decay of GLU is combined with production of NADH_2_ or NADPH_2_. Both these molecules are high energy compounds and its free energy corresponds approximately to the energy of 3 ATP molecules. The activity of GDH^21^ is regulated by allosteric inhibitors, ATP, NADH_2_ and by the activator, ADP, as well. Thus, this enzyme is active in the state of the lack of energy in the cell. It makes the biological sense. In the case of high energy level in the cell (high concentration of ATP, NAD(P)H_2_, and *α*KT), the synthesis of GLU should dominate. This would open the minicycle between GLU and *α*KT catalyzed by GDH^21^ and AAO^20^ and would lead to the dissipation of energy stored in NAD(P)H_2_. Thus, GDH^21^ is turned off. In the case of insufficient energy level in the cell the quick energy pathway from GLN and GLU is open. The result is the increase of GLU and GLN decay and the decrease in GLN levels in the blood. It causes the activation of muscle decay in order to maintain the GLN levels in the blood and provide the GLN to the cells that need it. The tissues that especially need the energy for the fight against the tumor and for repairing damages after chemo- and radiotherapy are the intestine epithelium and the immune system [6–21].

The gluconeogenesis is the second reason of quicker GLN metabolism in cancer patients, especially in the advanced state of the illness. It is known that cancers use the glucose as the main source of ATP, according to the so-called Warburg effect [22]. In the case of high glucose intake by the cancer, the glucose is restored in the liver from amino acids and GLN is the main source of amino acids in this process [23].

Thus, GLN is released from muscles in the periods of increased metabolic stress and the concentration of the intracellular GLN decreases by more than 50% [24, 25]. The resynthesis of GLN cannot be sustained on the exact level in these periods.

The suggested glutamine requirement after uncomplicated major operations, major injury, gastrointestinal malfunctions, and during cachexia is ~0.15–0.20 g glutamine per kg body weight. In patients with serious immune deficiency, after bone marrow transplantation, during episodes of sepsis, systemic inflammatory response syndrome, or multiorgan failure, the requirement is increased to ~0.3–0.5 g glutamine per kg body weight [5, 26–28].

It is difficult to provide this amount of GLN to the diet because generally the intake of natural GLN does not exceed 10 g. Thus, although the organism is capable of synthesizing the GLN, it should be treated as deficient nutrient in the periods of increased metabolic stress.

### 2.2. Metabolism of Glutamine in Cancer Cells

**Figure 2:**
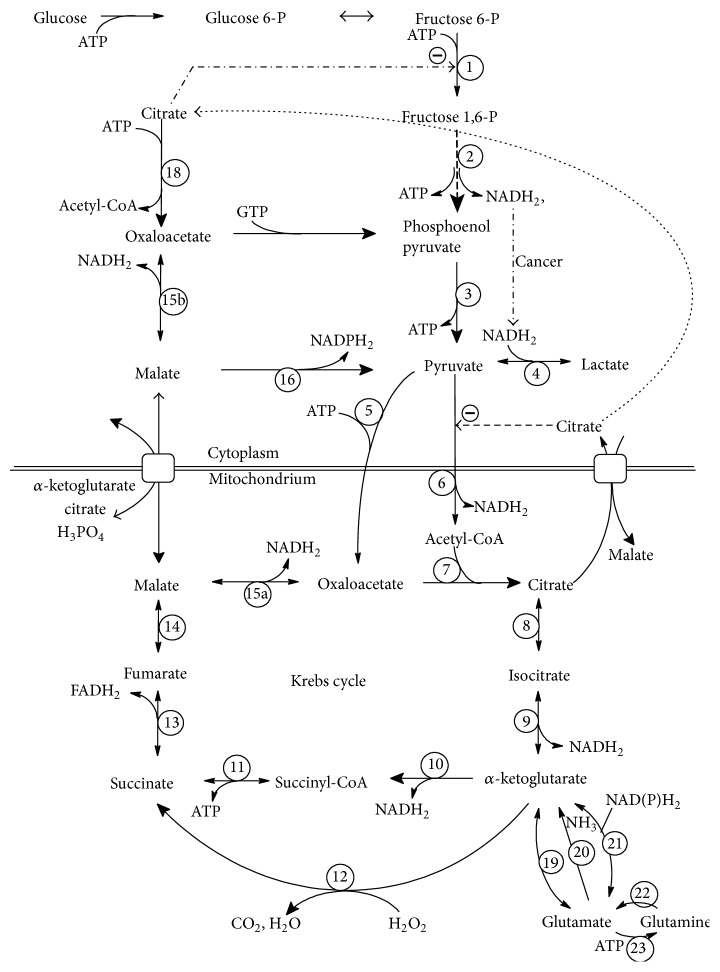
General scheme of glutamine (GLN) utilization in main metabolic pathways with details concerning ATP, NADH_2_, and NADPH_2_ production/utilization. PDHC^6^ catalyzing the reaction pyruvate→Acetyl-CoA is the only reaction that is able to reduce the total number of TCA molecules in the cell; thus, every GLN degradation pathway goes to pyruvate. Two pathways can be defined (see also Figure 3): (1) glutaminolysis (GLL): GLN→*α*KT→SC→malate→(to cytoplasm)→pyruvate and (2) reverse-TCA (R-TCA): GLN→citrate→(*to cytoplasm*)→oxaloacetate→malate→pyruvate. The balance of GLL pathway is +1 FADH_2_, +1 NADPH_2_, and +1 ATP (alternatively 0 ATP in the case of reaction 12). The balance of R-TCA pathway is −2 NADH_2_, −1 ATP, and +1 NADPH_2_. The alternative NAD(P)H_2_ from the reaction 21 is omitted. The conversion of Acetyl-CoA to malonyl-CoA in the fatty acid synthesis utilizes 1 additional ATP. Only one ATP molecule can be created directly during degradation of GLN in the GLL pathway (reaction 11). R-TCA utilizes rather ATP (for enzyme names, see enzyme abbreviation list).

The cancer cells differ significantly from the normal cells in the intensity of the main metabolic pathways. The most important differences consist in the following:

- higher level of the oxidative stress accompanying the metabolic malfunctions [25, 29–36],
- increased aerobic glycolysis and production of lactic acid (Warburg effect) [22],
- production of ATP mainly in the aerobic glycolysis process [22, 34, 37, 38],
- reduction of the activity of pyruvate dehydrogenase complex PDHC^6^ that converts pyruvate to mitochondrial Acetyl-CoA [34, 37, 39]* (the upper indices at the enzyme abbreviation point to the reaction numbers in Figures 1 and 2)*,
- reduction of intensity of TCA cycle due to reduced activity of some TCA cycle enzymes [29–31, 33, 40, 41],
- reduction of activity of the oxidative phosphorylation (OXPH) in the cytochrome chain [25, 29–31, 33],
- reduced activity of the pentose phosphate cycle and production of NADPH_2_ for fatty and nucleic acid synthesis mainly by the malic enzyme (ME^16^),
- high utilization of glutamine mainly for the production of NAPDH_2_ that is used mainly for fatty and nucleic acid synthesis and for restoration of the molecules of TCA cycle that leave mitochondria for different anabolic purposes of the intensively dividing cells [42, 43],
- high activity of ATP citrate lyase (ACL^18^) in the cancer cells that produce cytoplasmatic Acetyl-CoA due to higher demand of the cancer cells for the fatty acid synthesis [44, 45],
- increased activity of hypoxia inducible factor (HIF-1*α*) in cancer cells both in the hypoxic [46–48] and normoxic conditions [49].

Glutamine is the controversial diet supplement that can modify the metabolism in both cancer and normal cells. Different aspects of GLN point either to the potential disadvantages or to potential benefits of its supplementation in cancer patients. GLN is recently presented to be one of the main nutrients for cancer growth [42, 50]. The most important role consists in supplying the reduced hydrogen in the form of NADPH_2_. NADPH_2_ is next utilized by cancer cells mainly for the fatty and nucleic acids synthesis. GLN can be also the significant source for gluconeogenesis in the liver, being the source of glucose for the cancer.

On the opposite, many papers point to the positive role of GLN supplementation. Let us summarize the potential beneficial effects of GLN supplementation [23]:

1. stimulation of NK lymphocytes metabolism and intestine mucosa regeneration [12, 51–54];
2. reduction of the side effects of chemo- and radiotherapy and especially the reduction of intestinal mucosa injures [13–21, 55];
3. increase in some therapeutic effect of chemotherapy, among other things by the increased concentration of some drugs inside the tumor cells [15, 18].

In the next step, let us deal with these aspects that exhibit the potential slowing down effect on tumor growth, however, accompanied by increase in the resistance to starvation and/or oxidative stress:

(4) activation of the autophagy in the cancer cells due to the increased ammonia production. Autophagy is the process of self-digestion which makes it possible to recycle the cellular proteins and lipids into its metabolic precursors. This process promotes cell survival in the case of starvation or other metabolic stresses [42, 56];
(5) stimulation of the glutathione synthesis which inhibits the oxidative stress in healthy cells and contributes to the cancer cell growth inhibition [57–59]. It can make, however, the cancer cells more resistant to chemo- and radiotherapy [60].

In analyzing GLN intake as a possible positive or negative factor supporting the cancer growth and/or cancer treatment, one must take into consideration the differences between the metabolism of healthy and cancer cells [2]. The main problem of this analysis is the variety of metabolic changes characterizing different cancer types. Individual types of cancer metabolism should be analyzed with regard to the possible positive or negative effect of glutamine supplementation. Some methods of metabolic analysis of cancer cells are available [39, 61, 62].

At first, the division into two main groups of cancer types should be analyzed: cancers with the (a) normal and (b) reduced activity of pyruvate dehydrogenase complex (PDHC^6^). The activity of this enzyme is deteriorated in the majority of cancers [34, 37, 39]. Theoretical analysis of GLN degradation presented in this paper shows that the GLN supplementation may be beneficial especially in cancers with reduced PDHC^6^ activity.

## 3. Glutamine and Glutathione

Glutamine is significantly involved in the synthesis of glutathione (GSH)—the tripeptide that comprises three amino acids: glutamic acid, cysteine and glycine. This compound serves as a very important intracellular antioxidant and detoxication factor. Besides working as a scavenger of reactive oxygen species (ROS), GSH is involved in a variety of other metabolic functions such as DNA repair, activation of transcription factors, cell cycle regulation, modulation of calcium homeostasis, and regulation of enzyme activity. Most of these functions of GSH are related to its ability to maintain reduced cellular environment [60, 63]. Malignant diseases are accompanied by GLN deficiency and reduction of GSH in the host organism, which can be reversed by dietary GLN [58]. In addition, several reports suggested that the GSH constituent amino acids, including GLN, inhibit tumor promotion, at least in part, by their interference with GSH metabolism [57, 59].

The benefits of glutamine supplementation in cancers through the influence on GSH metabolism were broadly presented by Todorova et al. [60]. Tumor cells are shown to have higher concentration of reduced (active) form of GSH than the surrounding normal cells, which contributes to higher rate of cell proliferation and resistance to chemo- and radiotherapy. Therefore, selective tumor depletion of GSH presents a promising strategy in cancer treatment. Todorova et al. [60] have examined the effects of GLN on GSH levels in 7,12-dimethylbenz[*α*]anthracene- (DMBA-) induced mammary tumors and correlated the results with protein and mRNA expression of apoptosis-related proteins Bcl-2, Bax, and caspase-3 in tumor cells. The results have shown that GLN supplementation caused a significant decrease by 57% in tumor GSH levels and similar ratio GSH/oxidized GSH (GSSG) accompanied by upregulation of Bax and caspase-3 (apoptosis induced factors) and downregulation of Bcl-2 (apoptosis inhibiting factor). Bcl-2 is known to play a role in promoting cell survival and inhibition of apoptosis, while Bax, a member of the Bcl-2 family can induce apoptosis. Caspase-3 is the main apoptosis-induced enzyme. In the GLN supplemented group Bax mRNA has increased by 19% and caspase-3 mRNA by 30% and Bcl-2 mRNA decreased by 33%.

The importance of GSH depletion and reduction of GSH/GSSG ratio for stimulation of apoptosis has also been demonstrated in several* in vitro* models [59, 64–66]. GSH depletion was found to be necessary and sufficient to induce cytochrome c release, which is the key event in the apoptotic mitochondrial signaling pathway [67].

The next possible effect of GLN supplementation on the growth of cancer cells is the modulation of IGF-I and TGF-*β*1 concentrations in both cancer and normal cells. The proteins of insulin-like growth factor system (IGF) are known to play an important role in tumor genesis and inhibition of apoptosis. Transforming growth factor (TGF-*β*) is a cytokine involved in the process of cell migration, tumor vascularisation, and inhibition of cell proliferation. It is not explained if the observed effect of GLN supplementation on Bax, Bcl-2, capsace-3, IGF-I, and TGF-*β*1 levels is the direct effect of GLN or the intermediate effect of the altered GSH metabolism.

## 4. Glutamine and ATP Production in Cancer

Alterations in the cancer cell metabolism consist as a rule in the increased glycolysis [22], decreased TCA cycle activity [40, 41], and decreased oxidative phosphorylation (OXPH) [25] in mitochondria. The TCA produces NADH_2_/FADH_2_ and OXPH uses them for ATP production. Thus, ATP production in mitochondria is often deteriorated in cancer cells and the main source of ATP remains glycolysis [22]. The common feature in cancer is the overproduction of reactive oxygen species (ROS) in the OXPH chain that leads to the downregulation of the ATP production in mitochondria and to the oxidative stress [25, 29–33].

The analysis of GLN metabolism presented below takes into account its lack of ability to produce ATP, especially in the context of the deteriorated ATP production via TCA cycle and OXPH chain [68].

### 4.1. Relation between Glutamine and Krebs Cycle

In order to investigate the effects of GLN supplementation, let us analyze the decay paths for GLN. In Figure 1, let us analyze two reactions that metabolize reciprocal reactions between GLN and GLU. As discussed earlier, in the case of low ATP level in the cell, which takes place in the majority of cancers, one can expect that the equilibrium between these both reactions is strongly shifted to the left (GLU creation).

The activity of GA^22^ in the individual types of cancers is one of important features of cancer metabolism concerning GLN [69]. This enzyme is encoded by GLS and GLS2 gene [70]. Expression of GLS2 is necessary for cells to maintain GSH levels and silencing GLS2 increased ROS and oxidative damage of DNA. It is reported that gene GLS2 encoding “liver-type” isozyme of GA^22^ is highly expressed in normal adult liver but silenced in hepatocellular carcinomas [71, 72]. Thus, in the case of cancers having reduced activity of GA^22^, the increased oxidative stress in the cancer cells is expected. GLN supplementation is, however, not expected to reduce this oxidative stress and support the growth of the cancer cells. The benefits of GLN supplementation may potentially surpass disadvantages in this case.

Now, let us analyze relations between *α*KT and GLU. The crucial enzyme here is glutamate dehydrogenase (GDH^21^). As mentioned, this reaction relies on the metabolic state of the organism and the direction GLU→*α*KT dominates, due to the allosteric regulation. The activity of GDH^21^ is inhibited by ATP and NADH_2_ and activated by ADP. It means that this enzyme is active in the state of energy shortage in the cell opening the quick energy pathway from GLN. It results in the GLN decay and decrease in GLN levels in the blood. The low GLN level activates the muscle proteins decay in order to maintain the GLN levels in the blood. In the case of high GLN utilization by the advanced cancer, this process causes the cancer cachexia. The decay of GLN takes place, however, also in healthy cells. In particular, the intestine epithelium and immune system need GLN as a primary fuel and both these systems are crucial against tumor and repair damages after chemo- and radiotherapy [10, 12, 14, 15, 73–75].

The reactions catalyzed by AAO^20^, GDH^21^, and GA^22^ produce ammonia. Ammonia produced in these reactions inside the tumor plays a crucial role in autophagy regulation [42, 56]. GLN supplementation is expected to increase the ammonia production, especially in the case of cancers with normal activity of GA^22^. Stimulation of autophagy denotes potentially slower growth of the tumor and, at the same time, possibly higher resistance to chemo-/radiotherapy, starvation, and/or oxidative stress.

It is reported that in the case of glucose (GLC) deprivation, oxidation of GLN supports cell viability rather than its growth [76]. Thus, supplementation of GLN, especially accompanied by GLC withdrawal or glycolysis-inhibition therapy, may be treated as a potentially beneficial approach. An inverse process (*α*KT→GLU→GLN) occurs especially in muscles after the protein consumption. The reaction *α*KT→GLU is catalyzed by the transaminase (TA^19^) and it is connected with the shift of –NH_2_ pool from other amino acids into GLN. Thus, this pathway can take place especially in the abundance of other amino acids that can shift its –NH_2_ group to *α*KT, as it is carried out in muscles.

### 4.2. Glutamine versus Pyruvate Dehydrogenase Complex (PDHC^6^)

TCA cycle and oxidative phosphorylation (OXPH) are two factors that are essential for the effective aerobic ATP production.  Figure 2 shows the TCA cycle reactions, the final part of the glycolysis pathway, and additional reactions in the cytoplasm that are involved in the glucose and amino acids metabolism. One of the main changes in cancer metabolism observed in majority of cancers is the deterioration of the activity of pyruvate dehydrogenase complex (PDHC^6^) that catalyses the reaction: pyruvate→Acetyl-CoA. This inhibition causes the reduced ATP creation [22, 34, 37–40]. Cancers with reduced PDHC^6^ activity produce ATP mainly using glycolysis that is a significantly worse source of ATP than TCA + OXPH pathway. But also metabolic blocks of other TCA cycle enzymes or OXPH chain can lead to the deteriorated ATP production. Thus, the ATP level can be treated in many cases as an essential factor determining the velocity of tumor growth. Due to this observation, metabolism of the given nutrient should focus especially on its ability to support or inhibit ATP production.

In the analysis of cancer metabolism, it must be observed that one of the crucial points to be analyzed is the total number of TCA molecules in the cell (*N*_TCA_), counted both in mitochondria and in cytoplasm. *N*_TCA_ is supplied by the stream of GLC and amino acids, but only one reaction is able to reduce *N*_TCA_, namely, reaction catalyzed by PDHC^6^. Every other reaction presented in Figure 2 converts only the molecules of TCA without changing its total quantity in the cell. Thus, every considered pathway of GLN degradation must go to the pyruvate.

The alternative ways of pyruvate degradation are the reactions pyruvate→lactate and pyruvate→alanine and its successive excreting outside the cell. This process takes place in many cancer types and particularly in those which are characterized by the decreased PDHC^6^ activity. Thus, the remaining PDHC^6^ activity, LDH^4^ activity, and the ability to remove lactate outside the cell are crucial points determining the velocity of all metabolic paths lying before these reactions.

GLN is a molecule that must be metabolized to pyruvate. It means that, in the case of cancers with the deteriorated PDHC^6^, its degradation will not, in theory, occur quicker after supplementation, because GLN will “wait in line” to be metabolized by PDHC^6^ or LDH^4^. The GLN-“waiting in line” is, however, not expected in healthy cells which are characterized by normal PDHC^6^ activity.

In theory, another possibility for *N*_TCA_ reduction is entering the pentose phosphate pathway by conversion to glucose-6P. This process is, however, inhibited by high level of AMP being connected with low level of ATP. Those cancers with low PDHC^6^ activity are characterized by low ATP level since the effective energy production in mitochondria is not efficiently supplied with Acetyl-CoA. Thus, one can expect that the reduction of *N*_TCA_ by pentose phosphate pathway is nondominating.

The other possibility for *N*_TCA_ reduction is the conversion to some nonessential amino acid and its use in the protein synthesis. One should, however, remember that the limitation for the protein synthesis in the cancer cells depends mainly on the essential amino acids pool. The standard diet supplies both the essential and nonessential ones. GLN may support only the pool of nonessential ones. The conclusion may be drawn that this way is also not a dominating one. The possible negative influence of GLN supplementation may be expected and, however, is the case of simultaneous supplementation of GLN and essential amino acids only. Taking the essential amino acid pool into account, one can assume that the influence of GLN supplementation should be more effective if the essential amino acid pool would be simultaneously reduced in the diet.

## 5. Utilization of GLN

**Figure 3:**
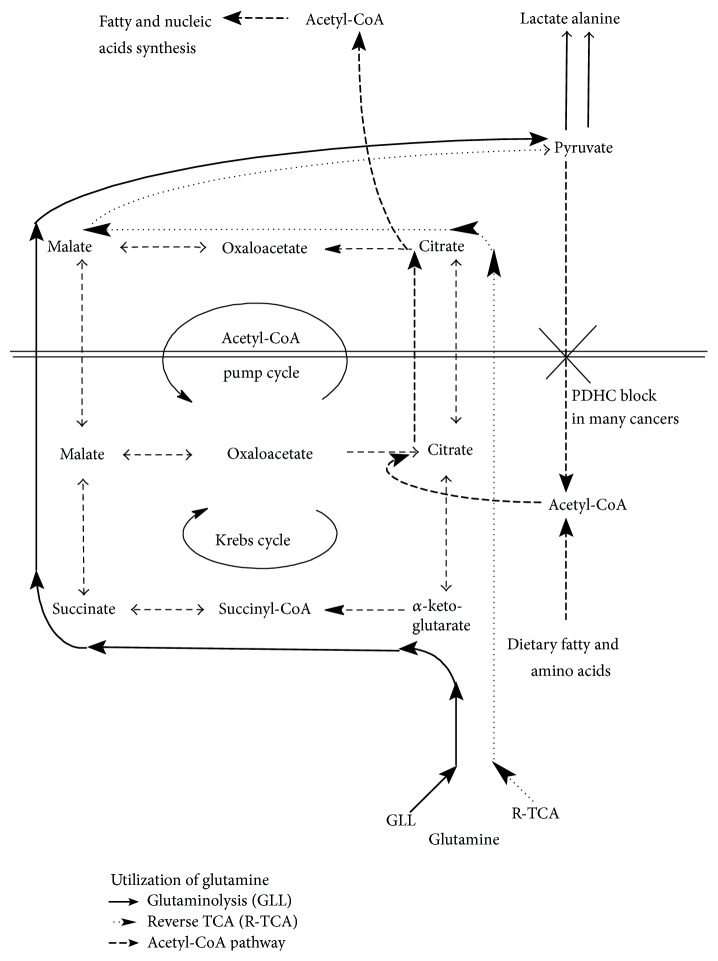
The schematic presentation of two glutamine metabolism pathways: glutaminolysis and reverse TCA and its connection with the Acetyl-CoA pump that transports Acetyl-CoA from mitochondria to cytoplasm. In the case of deteriorated PDHC^6^ activity as in majority of cancers, the carbon skeleton of GLN can be metabolized only to lactate or alanine. The other pathways are rather negligible.

The analysis of GLN utilization in cancer cells must be considered separately for the cancers possessing the reduced and normal activity of PDHC^6^ and it must be performed especially with respect to ATP and reduced hydrogen production. Two pathways of conversion GLN→pyruvate can be considered depending on the activity of different enzymes and physiological state of the cell. They are presented in Figure 3 where they are called* glutaminolysis *(GLL) and* reverse TCA *(R-TCA). The basal way is GLL. Malate is the molecule that leaves mitochondria and it is next converted to pyruvate. This pathway is, however, reduced in the case of decreased activity of TCA enzymes catalyzing the reactions between *α*KT and malate that are described in some types of cancer [48, 77, 78].

The alternative pathway is R-TCA that consists in the conversion of *α*KT to citrate. Citrate leaves mitochondria and it is converted in the cytoplasm through oxaloacetate to pyruvate. This pathway is combined with one cycle of Acetyl-CoA pump that transports Acetyl-CoA from the mitochondria to the cytoplasm. Acetyl-CoA can be defined as the third most important (together with ATP and NADPH_2_) crucial nutrient for the tumor growth. This pathway is, however, from *N*_TCA_ point of view, independent of Acetyl-CoA pump cycle which transports Acetyl-CoA to the cytoplasm and NADH_2_ from cytoplasm to mitochondria.

The source for Acetyl-CoA can be the pyruvate, dietary fatty acids, and amino acids. Pyruvate can be effectively converted to Acetyl-CoA only if the activity of PDHC^6^ is maintained. The process of Acetyl-CoA transport to the cytoplasm for the purpose of fatty and nucleic acid synthesis was analyzed in the subject literature [44, 45]. The current paper focuses rather on the importance of proportion ATP/NADPH_2_ in cancer cell because GLN supplementation seems not to influence the Acetyl-CoA pump. The amount of Acetyl-CoA appears important only in the case of cancers having deteriorated both PDHC^6^ activity and fatty/amino acid transport and/or their metabolism to Acetyl-CoA in mitochondria. On the other hand, it is possible that the Acetyl-CoA pump can be also deteriorated in some cases, making Acetyl-CoA the vital nutrient for the cancer growth. The crucial enzyme of Acetyl-CoA pump is cytoplasmatic ATP citrate lyase (ACL^18^). Its activity is often increased in cancer cells and its inhibition is proposed to be the target for cancer treatment [44, 45]. GLN can be the source of Acetyl-CoA only in the case of normal or close to normal activity of PDHC^6^ [43].

### 5.1. Glutaminolysis

Now, let us analyze in details, two GLN→pyruvate pathways. GLN enters TCA cycle before the *α*-ketoglutarate dehydrogenase complex (KGDHC^10^). It means that the proper activity of both KGDHC^10^, succinyl-CoA synthetase (SCS^11^), succinate dehydrogenase (SDH^13^), and fumarate hydratase (FH^14^) is necessary for GLN utilization inside the TCA cycle. This process (*glutaminolysis, *GLL; see Figures 2 and 3) is the basal pathway of GLN metabolism in the normal cells with the only exception that the pyruvate is metabolized to Acetyl-CoA and next enters the TCA cycle. Enzymes KGDHC^10^ and SDH^13^ are present only in mitochondria. Thus, the proper metabolism of GLN in the GLL pathway requires proper functioning of this part of TCA cycle in mitochondria.

GLN is able to provide directly in the GLL pathway only 1 molecule of ATP in the reaction of succinyl-CoA to succinate (SDH^11^). It is twice less than the amount of ATP produced from glucose (GLC) in the anaerobic glycolysis (2 ATP/1 GLC). The remaining reactions of GLN degradation produce energy through NADH_2_ (GDH^19^, KGDHC^10^, and MDH^15ab^), FADH_2_ (SDH^13^), or NADPH_2_ (GDH^19^, ME^16^). Moreover, the only molecule of ATP may not be created in the existence of the oxidative stress in cancer cell. Fedotcheva et al. [79] show that the nonenzymatic decarboxylation of *α*KT (reaction 12), pyruvate, and oxaloacetate induced by H_2_O_2_ results in the formation of succinate, acetate, and malonate, respectively. The only molecule of ATP may not be created in this situation. It is proved that many cancer cells (lung, breast, kidney, prostate, colon, liver, skin, thyroid, and bladder) present the existence of oxidative stress according to mtDNA mutations followed by impaired TCA cycles enzyme synthesis and/or OXPH chain [29–31, 33]. Thus, one can assume that the nonenzymatic decarboxylation without synthesis of the only ATP molecule may occur common in cancers.

Many experiments point to the decreased activity of both different TCA enzymes and OXPH in cancer cells [29–31, 33]. Blocking or reducing some of these enzymes makes it difficult to metabolize GLN in the GLL pathway.

The decreased activity of KGDHC^10^ catalyzing the reaction *α*KT→succinyl-CoA can be expected in tumors having deteriorated activity of PDHC^6^ since PDHC^6^ and KGDHC^10^ are twin enzymatic complexes that can undergo similar regulatory processes [80].

The additional effect can be connected with the nonenzymatic conversion of oxaloacetate to malonate. Malonate is known to be mitochondrial toxin [81] considered as the competitive inhibitor of SDH^13^ and trigger of superoxide radicals [82]. The malonate degradation in the cell depends on the concentration of ATP and Mg^2+^ [83]. The concentration of these two compounds is decreased in the majority of cancers causing possible further decrease in activity of this part of TCA, due to possible increased malonate concentration in the cancer cell.

It can be observed that the presented pathway of GLN decay does not produce significant ATP amounts in the cancer cells. NADH_2_ and FADH_2_ can be the source of ATP only after they enter the OXPH chain. The decrease in OXPH protein contents, respiratory chain activities, and mitochondrial DNA amounts in cancer are well evidenced, particularly in CCRCs [78, 84–86] but also in other types of cancers [87–90]. Thus, the ability of GLN to support ATP production is expected to be strongly reduced in the cancers having lowered OXPH activity.

However, in the case of cancers with OXPH chain and GLL pathway working properly, GLN supplementation may support ATP production which is generated from NADH_2_ and FADH_2_ in the OXPH chain. It can take place, however, if the twin enzyme complexes (PDHC^6^ and KGDHC^10^) have different activities: PDHC^6^ is inactive and KGDHC^10^—active.

Concluding, mainly cancers having deteriorated OXPH activity or *α*KT→malate part of TCA cycle can be treated as potentially surpassing the benefits over the disadvantages while supplementing GLN.

### 5.2. Reverse TCA

The GLN metabolism can take place in the case of decreased activity of KGDHC^10^, SCS-A^11^, SDH^13^, or FH^14^. GLN can be metabolized to citrate in the R-TCA reactions (IDH^9^ and Aconitase^8^). Next, citrate is transported to the cytoplasm (see Figures 2 and 3). Here, citrate inhibits two enzymes: phosphofructokinase (PFK^1^) and pyruvate dehydrogenase complex (PDHC^6^)—the key enzymes of glycolysis. Thus, in the case of cancers possessing deteriorated GLL pathway, GLN supplementation can slow down the glycolysis (the main source of ATP) in some types of tumor by the agency of citrate [91]. This phenomenon is mainly expected in the case of tumors possessing deteriorated GLL pathway which forces conversion of GLN to citrate. Next, the citrate is metabolized in the cytoplasm by ATP citrate-lyase (ACL^18^) to Acetyl-CoA and oxaloacetate. This reaction uses 1 molecule of ATP. Oxaloacetate can be metabolized to malate (MDH^15b^) and next by using the NADP-malic enzyme (ME^16^) to pyruvate. Both products of ME^16^ (NADPH_2_ and Acetyl-CoA) can be the substrates to fatty and nucleic acid synthesis. The use of Acetyl-CoA for this process utilizes one additional molecule of ATP for its activation to malonyl-CoA [92]. There is, however, also a disadvantage of this pathway. The reactions catalyzed by cytoplasmatic MDH^15b^ and ME^16^ convert cytoplasmatic NADH_2_ to NADPH_2_, which leads to the additional glycolysis reaction producing NADH_2_ without accompanying lactate creation that produces extra 2 ATP molecules. Summing up, the ATP production balance of R-TCA pathway is close to zero. The described process occurs particularly in the case of deteriorated activity of KGDHC^10^, SCS-A^11^, SDH^13^, or FH^14^. The normal pathway of TCA cycle cannot follow in that case.

GLL and R-TCA pathways are the only two fundamental pathways for GLN conversion to pyruvate. The other pathways could be considered as the connections of one of the above defined pathways and of another cycle of reaction. Namely, if GLL pathway was joined with Acetyl-CoA pump cycle (see Figure 3), then the following pathway could be obtained: GLN→*αKT*→*SC*→*malate*→*oxaloac*→*citrate*→(*transport to cytoplasm*)→*oxaloac*→*malate*→*pyruvate.*

The analysis of GLN metabolism presented above leads to the main conclusion that GLN cannot be, in practice, the significant source of ATP. In the case of lowered ATP level in the cancer cell, the supplementation of GLN does not supply the cell significantly in ATP and the supplementation of GLN may be beneficial rather than disadvantageous. It should be, however, stressed that the beneficial effect is expected particularly if the ATP reduction is supported by accompanying glucose withdrawal in the diet and/or glycolysis inhibition therapy that decreases ATP being a crucial metabolic substance for tumor growth.

## 6. Glioblastoma

All considerations presented in the former section concerned mainly cancers with the deteriorated PDHC^6^. Some cancers possess, however, the normal or close to normal activities of PDHC^6^ and OXPH chain. The example is the glioblastoma multiforme (GM) [32, 43] that shows normal activity of the TCA cycle and only partially deteriorated ability to produce the ATP from fatty acids in the OXPH chain [2]. The activity of pyruvate kinase PC^5^ in place of PDHC^6^ is deteriorated in these cells. The escape of citric acid from mitochondria for fatty acid synthesis (Acetyl-CoA pump) is also observed. The oxaloacetate used for citric acid synthesis in the mitochondria is derived to a greater extent from the GLN and the Acetyl-CoA from glucose in this case. It must be observed that, due to proper activity of PDHC^6^, pyruvate is able to enter TCA cycle and to be the source for ATP production [93]. Thus, it can be concluded that the supplementation of GLN is rather not recommended in this case because GLN can be the source of both ATP, NADPH_2_, and Acetyl-CoA. DeBerardinis et al. [43] showed GM by using the ^13^C NMR spectroscopy that in the case of abundance of both GLN and GLC about 1/3 of Acetyl-CoA for fatty acid synthesis comes from GLN and about 2/3 from GLC. This is possible due to maintained PDHC^6^ activity.

Since the number of TCA molecules in the cell is reduced by PHDC^6^ and not increased by PC^5^, GM cells need to be continuously supplied with TCA molecules for maintaining the metabolic pathways. In this case, the main source is GLN as the most important amino acid in blood that can be converted to some TCA molecule.

Many cancers lower ATP production in the case of GLC withdrawal [39, 94] but not the GM [32]. In the case of glucose abundance, about 84% of GLC is metabolized to lactic acid, 9% is metabolized to alanine, and 5% is metabolized in the OXPH chain [43]. This proportion reflects the typical Warburg effect. In the case of GLC withdrawal, GM stops the ATP production in the glycolysis pathway but follows the OXPH chain and the total ATP level does not decrease in the cancer cell. This process is combined with high ROS production in the OXPH chain and the GM cells start to die out due to the increased oxidative stress [32]. Thus, the supplementation of GLN as the source of NADH_2_/FADH_2_ for the impaired OXPH chain producing many ROS can be potentially beneficial in this case. It should be, however, accompanied with the strong carbohydrate reduction in the diet and/or with the glycolysis inhibition therapy and/or with the increase in amino- and fatty acids in the diet that supports TCA cycle and in this way the oxidative stress in the cell. The detailed “*in vivo*” analysis of this approach must be, however, performed to answer if the destroying effect of the oxidative stress overcomes the tumor growth stimulation.

## 7. Glutamine and HIF-1*α*

It is proved that many tumors show the overexpression of hypoxia inducible factor (HIF-1*α*). It is reported that HIF-1*α* can be activated by a number of other oncogenes even under normoxic conditions [49]. Thus, the increased activity of HIF-1*α* is probably a feature of many tumors.

HIF-1*α* is degraded by one of three different HIF prolyl hydroxylases. They are members of a superfamily of iron and *α*-ketoglutarate-dependent dioxygenases [95, 96]. The overexpression of HIF-1*α* occurs due to the inhibiting activity of accumulated succinate (SC) on the HIF prolyl hydroxylases. In other words, HIF-1*α* activation is stimulated by lowered *α*KT/SC ratio.

The overexpression of HIF-1*α* leads to the increased transcription of genes encoding glycolysis enzymes like aldolase^2^, pyruvate kinase^3^, and LDH-A^4^ [46–48]. The target is also the pyruvate dehydrogenase kinase that inactivates the PDHC^6^. The inhibition of PDHC^6^ by HIF-1*α* causes the accumulation of pyruvate and lactate. If the removal of lactate from the cancer cell is not sufficient then the other probable consequence of this inhibition is the accumulation of preceding metabolites like malate, oxaloacetate, fumarate, and alternatively the succinate. In the case of GLN deficiency (and successive deficiency of *α*KT), this situation can lead to the decreased ratio of *α*KT/SC and further stabilization of HIF-1*α*.

GLN is a quick source of *α*KT in the cancer cell. The inhibiting effect of *α*KT supplementation on HIF-1*α* activity is described by Matsumoto et al. [97]. The* in vitro* antiproliferative effect of *α*KT on some kinds of tumors was also presented by Brzana et al. [98]. Thus, one of the possible positive effects of GLN supplementation can be making *α*KT/SC ratio increased in cancer cells which can reduce the overexpression of HIF-1*α*.

Many other beneficial effects of *α*KT supplementation are described by Harrison and Pierzynowski [99]. It suggests that some of the benefits of GLN supplementation are probably obtained by the agency of *α*KT. The reduction of HIF-1*α* should lead to the reduction of overexpressed glycolysis and to restoration to some degree of the PDHC^6^ activity. It is recently shown that restoration of PDHC^6^ activity through dichloroacetate in cancer cells can promote the apoptosis of cancer cells [37]. On the other hand, activation of PDHC^6^ can support the energy production in the cancer cells and the effect of GLN could be negative if the apoptosis caused by HIF-1*α* inhibition did not occur. However, the detailed analysis of this problem was not found.

## 8. Gluconeogenesis

Main problem related to the GLN intake by cancer patients is gluconeogenesis. Gluconeogenesis is one of the reasons for quicker GLN metabolism in cancer patients, especially in severe stages of the illness. It is known that cancers use the glucose as the main source for ATP production [22]. In the case of high glucose intake by the cancer, glucose is restored in the liver from amino acids and GLN is the main source for this process. Thus, there is a potential danger of GLN supplementation to produce glucose for ATP production in the tumor. The main aim of GLN supplementation in cancer patients is to support the immunological system, intestine tract, and glutathione synthesis and inhibit the cancer cachexia. This problem can be probably solved by experiments that will monitor the concentration of GLC and GLN in the plasma in different dietary and/or therapy conditions. Most effective doses and application time of GLN should be found in order to substantially support the organism rather than gluconeogenesis and cancer. The optimal effect of GLN supplementation is expected when it is accompanied by gluconeogenesis and/or glycolysis inhibition to slow down ATP production. Supplementation of GLN seems to make sense if the concentration of both GLN and GLC in plasma is low. GLN supports, in this case, the regeneration of the organism without significant support of tumor growth. Support for the intestine tract (e.g., after chemo- or radiotherapy) should be made orally, but supporting the immunological system and other healthy tissues should be made rather intravenously to omit the effect of first GLN passage through the liver. The elementary doses of GLN should be probably small enough to be consumed by healthy tissue in particular rather than converted by liver to GLC.

## 9. Summary

Based on the analysis of GLN metabolism, it can be concluded that if the OXPH chain is deteriorated, GLN cannot be an effective source of ATP for the cancer cell regardless of the metabolic pathway. The benefits of GLN supplementation should be probably more significant if they are accompanied by significant carbohydrate restrictions in the diet and by glycolysis and/or gluconeogenesis inhibition therapy, which will reduce the ATP level in the cancer cells that have the deteriorated mitochondria, but not in the normal cells with correctly functioning mitochondria. GLC and GLN monitoring in plasma is recommended in order to find the optimal doses and intervals of GLN that minimize gluconeogenesis effects in the liver. On the other hand, the apoptosis induction effect of GLN supplementation via HIF-1*α* inhibition is also possible. Supporting the intestine tract, immunological system and glutathione synthesis by GLN are especially expected to be beneficial for patients undergoing chemo- and radiotherapy. The supplementation may be highly effective after the chemo-/radiotherapy to avoid any therapy resistance effects. The other problem of GLN supplementation is its inhibition of cancer cachexia. GLN can be considered to be applied as a part of palliative care.

The dose dependent effect is expected to be strongly nonlinear. In the case of small doses, the positive supplementation effect seems to depend on the reduction of GLN deficiency in healthy cells and supporting the intestine epithelium and immunological system. Medium doses are expected to support cancer metabolism rather than the organism itself. Big doses may be considered to induce HIF-1*α* inhibition that could activate PDHC^6^ and, next, apoptosis. *α*KT supplementation can be considered as an alternative approach that is expected to exhibit all effects of GLN with the exceptions concerning ammonia induced autophagy and promotion of GSH synthesis [100].

The following individual cancer metabolism features are very important in analyzing the effect of GLN supplementation: PHDC^6^ activity, individual TCA enzymes activity profile, malonate concentration, functioning of OXPH chain, oxidative stress, and HIF-1*α* activity.

## Abbreviations

### Enzymes (The Upper Indices Point to the Reaction Numbers in Figures 1 and 2)

AAO^20^: l-Amino acid oxidase
ACL^18^: Citrate-lyase
FH^14^: Fumarate hydratase
GDH^21^: Glutamate dehydrogenase
GA^22^: Glutaminase
GS^23^: Glutamine synthase
IDH^9^: Isocitrate dehydrogenase
KGDHC^10^: Alfa-ketoglutarate dehydrogenase complex
LDH^4^: Lactate dehydrogenase
MDH^15^: Malate dehydrogenase
ME^16^: Malic enzyme
PC^5^: Pyruvate carboxylase
PDHC^6^: Pyruvate dehydrogenase complex
PFK^1^: Phosphofructokinase
SCS-A^11^: Succinyl-CoA synthase
SDH^13^: Succinate dehydrogenase
TA^19^: Transaminase.

### Other Terms

*α*KT: Alfa-ketoglutarate
GLC: Glucose
GLN: Glutamine
GLU: Glutamate
GM: Glioblastoma multiforme
GSH: Glutathione
HIF-1*α*: Hypoxia inducible factor
OXPH: Oxidative phosphorylation
SC: Succinate
TCA: Tricarboxylic acid cycle.
